# Agranulocytosis Associated With Psychiatric Polypharmacy: Lessons Learned From a Clinical Case

**DOI:** 10.7759/cureus.56701

**Published:** 2024-03-22

**Authors:** Nicole Ann E Villa, Dragos G Pausescu, Eduardo D Espiridion

**Affiliations:** 1 Psychiatry, Drexel University College of Medicine, Wyomissing, USA; 2 Psychiatry, Reading Hospital-Tower Health, West Reading, USA; 3 Psychiatry, West Virginia School of Osteopathic Medicine, Lewisburg, USA; 4 Psychiatry, Drexel University College of Medicine, Philadelphia, USA; 5 Psychiatry, Philadelphia College of Osteopathic Medicine, Philadelphia, USA

**Keywords:** polypharmacy management, complex psychiatric conditions, drug interactions, agranulocytosis, psychiatric polypharmacy

## Abstract

Psychiatric polypharmacy involves the use of two or more psychotropic medications to manage a mental and emotional condition. The prevalence of psychotropic polypharmacy has been increasing since the 1990s and has been attributed to the rise in multiple psychiatric conditions presenting in one patient. However, as the prevalence of polypharmacy increases to maximize therapeutic advantages, so does the adverse effect profile of those drugs used in combination, leading to very life-threatening effects such as agranulocytosis. Thus, we report a case of agranulocytosis secondary to polypharmacy in a patient with a history of multiple complex psychiatric conditions.

The patient is a 20-year-old female with a past medical history of major depressive disorder, borderline personality disorder, post-traumatic stress disorder, anxiety disorder, hypothyroidism, and ulcerative colitis. Her psychiatric conditions were managed with multiple medications including chlorpromazine, and clozapine was recently added a month prior to admission. Upon admission, the patient was hemodynamically stable and febrile, with complaints of generalized body aches and myalgia. Laboratory results showed profound leukopenia with a white blood cell count of 1.0x10^3^/uL and a neutrophil number of 0.02x10^3^/uL. The patient was admitted to the hospital for neutropenic sepsis and was aggressively treated with intravenous antibiotics. Her clozapine and chlorpromazine were discontinued. In this report, we discuss the association between chlorpromazine and clozapine use and agranulocytosis, emphasizing the importance of regular monitoring and heightened awareness for patients on these medications. This case also underscores the necessity for cautious polypharmacy medication management in individuals with complex psychiatric conditions, highlighting the potential life-threatening consequences of polypharmacy in this population.

## Introduction

Psychiatric polypharmacy refers to the practice of prescribing multiple psychiatric medications to a single patient. This trend is common in the medical field because of the complexity of the clinical presentations of some psychiatric conditions. It aims to provide better disease management and symptom relief for patients whose psychiatric symptoms are inadequately managed by monotherapy [1]. The prevalence of any-class and multi-class psychotropic polypharmacy has been growing steadily, from 21.2% and 18.8% in 1999-2000 to 27.3% and 24.4% in 2009-2010, respectively [2]. This rise in polypharmacy is often justified in managing complex psychiatric disorders, such as treatment-resistant depression or bipolar disorder [3,4]. Some patients may be prescribed multiple medications from the same class like two or more antipsychotics. The complex nature of such diseases often requires a treatment plan tailored to the patient in order to provide optimal symptom relief. For patients with schizophrenia or major depressive disorder (MDD) that has not responded to monotherapy, adding another medication may be considered to achieve better symptom control.

As early as 1993, monotherapy has been described to inadequately control psychiatric manifestations and to develop tolerance to once-effective medications [5]. It is for this reason that clinicians explore combination therapies for patients they cannot otherwise control on a single medication or who are unable to achieve a level of symptom control that allows the patient to function on a daily basis. The increase in polypharmacy use can also be attributed to the rise in multiple psychiatric conditions presenting in one patient, such as MDD with features of anxiety or bipolar disorder [6]. For this reason, clinicians attempt trials of medications to achieve the best outcome for the patient, at times using drugs for off-label use and achieving very desirable outcomes. However, as the prevalence of polypharmacy increases in an attempt to harvest therapeutic advantages, so does the adverse effect profile of those drugs used in combination, leading to very life-threatening effects such as agranulocytosis. We report a case of agranulocytosis secondary to polypharmacy in a patient with a history of multiple complex psychiatric conditions. The main purpose of this study is to investigate the association between psychiatric polypharmacy and agranulocytosis especially since agranulocytosis can lead to serious or life-threatening infections. 

## Case presentation

The patient is a 20-year-old female with a past medical history of MDD, borderline personality disorder, post-traumatic stress disorder, anxiety disorder, hypothyroidism, and ulcerative colitis. She was transferred to the hospital from a psychiatric facility, with a presentation of weakness, dizziness, myalgia, and fever. Psychiatry was consulted to evaluate the patient for medication management. The patient had been a resident of a psychiatric facility for the past year, where she has a long history of depression with a sad mood, anhedonia, low energy, feelings of emptiness and worthlessness, as well as recurrent suicidal ideation. She has a long psychiatric history with multiple suicide attempts and self-injury by cutting. For the management of her psychiatric conditions, she was previously prescribed chlorpromazine 25 mg three times a day with clozapine 100 mg daily being added a month prior to admission. In addition, the patient was also prescribed topiramate, lamotrigine, lithium, bupropion, and citalopram. 

Upon admission, the patient was hemodynamically stable with a recorded fever of 101.3°F and was complaining of generalized body aches and muscle pains. Laboratory results were significant for profound leukopenia with a white blood cell (WBC) count of 1.0x10^3^/uL and a neutrophil number of 0.02x10^3^/uL. There is anemia as well as thrombocytosis (Table 1 and Table 2). There was discussion about a bone marrow biopsy, but the patient opted not to have it done during this admission. Chest X-ray and abdominal and pelvic CT scans showed no apparent sources of infection. Viral panel for flu/RSV/COVID tests, blood cultures, and urinalysis were negative for infection, and the patient was placed on neutropenic precautions. The patient was admitted to the hospital for neutropenic sepsis and was aggressively treated with intravenous antibiotics. During her hospitalization, she was administered doses of filgrastim 800 mcg, and her clozapine and chlorpromazine were discontinued. The patient's WBC count and neutrophil number gradually improved during admission. Her somatic symptoms improved, and she agreed to a hematologist follow-up in two weeks. On discharge, the patient was medically stable and returned to the psychiatric facility with a normal WBC count. 

**Table 1:** Other laboratory results on admission were within normal limits BUN: blood urea nitrogen

Basic metabolic panel	Results	Normal range
Sodium	136 mmol/L	136-145 mmol/L
Potassium	3.9 mmol/L	3.5-5.1 mmol/L
Chloride	105 mmol/L	98-107 mmol/L
Anion gap	9 mmol/L	5-12 mmol/L
BUN	9 mg/dL	7-25 mg/dL
Creatinine	0.89 mg/dL	0.6-1.3 mg/dL
Magnesium	1.9 mg/dL	1.9-2.7 mg/dL

**Table 2:** CBC results on presentation to the emergency department CBC results were significant for profound leukopenia with a white blood cell count of 1.0x10^3^/uL and a neutrophil number of 0.02x10^3^/uL CBC: complete blood count

CBC	Results	Normal range
White blood cell	1.0x10^3^/uL	4.8-10.8x10^3^/uL
Red blood cell	3.97x10^6^/uL	4.0-5.4x10^6^/uL
Hemoglobin	10.7 g/dL	12.0-16.0 g/dL
Hematocrit	32.4%	35.0-47.0%
Platelets	503x10^3^/uL	130-400x10^3^/uL
Lymphocyte number	0.94x10^3^/uL	0.7-5.2x10^3^/uL
Lymphocyte percent	92.2%	-
Monocyte number	0.06x10^3^/uL	0.1-1.3x10^3^/uL
Monocyte percent	5.9%	-
Neutrophil number	0.02x10^3^/uL	2.0-8.0x10^3^/uL
Neutrophil percent	1.9%	-

## Discussion

The use of polypharmacy exists in several categories, as described by the National Association of State Mental Health Program Directors (NASMHPD). Same-class polypharmacy refers to the use of more than one medication from the same class, for instance, the use of two selective serotonin reuptake inhibitors in a case of depression. Multi-class polypharmacy, on the other hand, is the use of full therapeutic doses of more than one medication from different classes for the same symptom cluster. An example is the use of valproate along with an atypical antipsychotic, such as olanzapine, for the treatment of mania. The use of one medication to treat the side effects of another medication from a different class is described as adjunctive polypharmacy such as using trazodone for insomnia caused by bupropion. Augmentation polypharmacy refers to the use of one medication at a lower-than-normal dose along with another medication from a different class in a full therapeutic dose for the same symptom cluster, for instance, the addition of a low dose of haloperidol in a patient responding partially to risperidone. Augmentation polypharmacy is also used to refer to the addition of a medication that would not be used alone for the same symptom cluster, such as the augmentation of antidepressants with lithium or thyroid hormones. Finally, total polypharmacy is the total count of medications used in a patient or the total drug load [7]. 

In the case presented, the patient received multi-class psychotropic polypharmacy in an attempt to better control her complex mood disorders, which led to her hospitalization and the subsequent diagnosis of agranulocytosis. The use of polypharmacy with psychotropic drugs in psychiatric facilities has been common practice as early as 1987 and implemented as a means to avoid emergent monotherapy treatment resistance [5,8]. The adverse effects of these medications have been well studied individually and as polypharmaceutical strategies for complex mood disorders [9,10]. 

Of the drugs that the patient was prescribed, chlorpromazine and clozapine have the deepest literature pool of causing agranulocytosis among patients of all ages. However, topiramate, lamotrigine, and bupropion are also associated with a possibility of agranulocytosis to a lesser extent than the previously mentioned medications [11]. Literature dating back to 2009 confirms the association between agranulocytosis and concomitant use of chlorpromazine and clozapine. While the literature has indicated a potential dose-dependent relationship, this mechanism of action remains incompletely understood [9,12,13]. Furthermore, the routine monitoring of blood tests before and during the administration of these medications has provided clinicians with the best chance to observe and intervene if neutropenia begins to develop prior to a cascade effect into agranulocytosis. The prevalence of agranulocytosis in those patients taking clozapine is 0.9%; however, neutropenia presents at 3.8% with its peak incidence a month after administration [13]. It is possible that the cumulative effect of multiple drugs, each with the potential to lower WBC count, synergized and led to the agranulocytosis that necessitated the patient's hospitalization. However, due to the large number of drug combinations, the desire to find relief for the patient, and the off-label use of psychotropic medications, predicting these adverse effects and drug-on-drug interactions becomes increasingly challenging for clinicians [14]. 

The complex landscape of polypharmacy and the lack of double-blind clinical trials make it almost impossible to predict their efficacy in combination and drug-on-drug interactions [15]. Therefore, clinicians prescribing the medication are presented with the increased burden of controlling multiple and complex psychiatric disorders, finding the medication that provides a patient with the most relief from their mood disturbances, and hoping side effects from polypharmacy do not arise. For this reason, recent studies have revisited testing on monotherapy regimens as a means to avoid unwanted and synergized side effect profiles with the hopes of providing the efficacy of polypharmacy [16,17]. Studies have also called for the development of guidelines to better understand polypharmacy for those cases where monotherapy is not adequate to control multiple psychiatric conditions [2]. 

Attempts have been made to link genomic predispositions to agranulocytosis and the medication that this patient has been prescribed. However, sample sizes and variability of medication dosing make it difficult to come to a concrete conclusion when compounded by polypharmacy. However, clozapine and chlorpromazine are both associated with a high risk of agranulocytosis (0.9% and 0.13%, respectively) [18]. Furthermore, their effects are believed to be dose-dependent as the bioactivation of clozapine leads to a buildup of chemically reactive nitrenium ions [19]. Furthermore, chlorpromazine can indirectly affect cell division by inhibiting enzymes involved in cellular DNA synthesis, such as DNA polymerase, thymidylate kinase, and RNA polymerase [20]. Presumably, the combination achieved by a buildup of chemically reactive ions and the inhibition of cellular DNA synthesis, through the simultaneous use of both medications, leads to neutropenia and, in severe cases such as the one described above, agranulocytosis. 

This patient's agranulocytosis was discovered in a timely manner, and the appropriate step of discontinuing the offending medications while ruling out sources of infection was made before more serious clinical problems arose. Drug-related blood dyscrasias leading to agranulocytosis carry with them a 5-10% mortality rate in Western countries [18]. For this reason, clinicians should proceed cautiously when prescribing these medications on their own and in combination, as neutrophil counts before and during the administration of these drugs can monitor dropping neutrophil counts before a patient's neutrophil number reaches zero. 

## Conclusions

This case showcases the balance needed to minimize the risks of adverse effects while maximizing therapeutic benefits for patients on multiple psychiatric medications. The escalating prevalence of polypharmacy reflects the changes being made in psychiatric care, where it is driven by the increasing complexity of co-existing psychiatric conditions in individual patients. However, as shown in the presented case of agranulocytosis secondary to polypharmacy, the pursuit of therapeutic optimization needs to be accompanied by regular monitoring and personalized medication management. Clinicians must remain watchful of the potential consequences of drug interactions, particularly with medications such as chlorpromazine and clozapine known to predispose patients to agranulocytosis. Heightened awareness, regular monitoring, and individualized treatment plans are crucial in ensuring the safety and well-being of patients with complex psychiatric conditions on polypharmacy regimens. 

## References

[1] Fountoulakis KN (2010). An update of evidence-based treatment of bipolar depression: where do we stand?. Curr Opin Psychiatry.

[2] Soria Saucedo R, Liu X, Hincapie-Castillo JM, Zambrano D, Bussing R, Winterstein AG (2018). Prevalence, time trends, and utilization patterns of psychotropic polypharmacy among pediatric Medicaid beneficiaries, 1999-2010. Psychiatr Serv.

[3] Si T, Wang P (2014). When is antidepressant polypharmacy appropriate in the treatment of depression?. Shanghai Arch Psychiatry.

[4] Amerio A, Russo D, Miletto N (2021). Polypharmacy as maintenance treatment in bipolar illness: a systematic review. Acta Psychiatr Scand.

[5] Reus VI (1993). Rational polypharmacy in the treatment of mood disorders. Ann Clin Psychiatry.

[6] McIntyre RS, Jerrell JM (2009). Polypharmacy in children and adolescents treated for major depressive disorder: a claims database study. J Clin Psychiatry.

[7] Kukreja S, Kalra G, Shah N, Shrivastava A (2013). Polypharmacy in psychiatry: a review. Mens Sana Monogr.

[8] Muijen M, Silverstone T (1987). A comparative hospital survey of psychotropic drug prescribing. Br J Psychiatry.

[9] Malhotra AK, Litman RE, Pickar D (1993). Adverse effects of antipsychotic drugs. Drug Saf.

[10] Apter JT, Kushner SF, Woolfolk RL (1994). Bupropion/nortriptyline combination for refractory depression. Ann Clin Psychiatry.

[11] Ahn YM, Kim K, Kim YS (2008). Three cases of reversible agranulocytosis after treatment with lamotrigine. Psychiatry Investig.

[12] Stephan F, Podlipski MA, Kerleau JM, Petit M, Guillin O (2009). Chlorpromazine-induced agranulocytosis: a case report [Article in French]. Encephale.

[13] Legge SE, Walters JT (2019). Genetics of clozapine-associated neutropenia: recent advances, challenges and future perspective. Pharmacogenomics.

[14] Moeller KE, Din A, Wolfe M, Holmes G (2016). Psychotropic medication use in hospitalized patients with borderline personality disorder. Ment Health Clin.

[15] Mojtabai R, Olfson M (2010). National trends in psychotropic medication polypharmacy in office-based psychiatry. Arch Gen Psychiatry.

[16] Saah T, Garlow SJ, Rapaport MH (2014). Provide optimized antidepressant monotherapy with multiple drugs before considering antidepressant polypharmacy. Shanghai Arch Psychiatry.

[17] Yee MR, Espiridon E, Oladunjoye AO, Millsaps U, Harvey N, Vora AH (2021). The use of clozapine in the serious mental illness patients enrolled in an assertive community treatment program. Cureus.

[18] Flanagan RJ, Dunk L (2008). Haematological toxicity of drugs used in psychiatry. Hum Psychopharmacol.

[19] Pirmohamed M, Park K (1997). Mechanism of clozapine-induced agranulocytosis : current status of research and implications for drug development. CNS Drugs.

[20] Pisciotta AV (1969). Agranulocytosis induced by certain phenothiazine derivatives. JAMA.

